# Peritoneal Dialysis As Salvage Therapy in a High-Risk Patient With Failed Hemodialysis Access: A Case Report

**DOI:** 10.7759/cureus.86681

**Published:** 2025-06-24

**Authors:** Selena Gajić, Marko Baralic, Ana Bontic, Aleksandar Sic, Aleksandra Kezic

**Affiliations:** 1 Nephrology, University Clinical Centre of Serbia, Belgrade, SRB; 2 Internal Medicine, Faculty of Medicine, University of Belgrade, Belgrade, SRB; 3 Nephrology, University Clinical Center of Serbia, Belgrade, SRB; 4 Neurology, School of Medicine, University of Belgrade, Belgrade, SRB

**Keywords:** bacteriemia, central venous catheter infection, end-stage kidney disease, hd (hemodialysis), hemodialysis access, peritoneal dialysis (pd)

## Abstract

Peritoneal dialysis (PD) is often contraindicated in patients with extensive prior abdominal surgeries due to the risk of adhesions, catheter malposition, and poor dialysis efficacy. We present a complex case of a 64-year-old male with end-stage kidney disease (ESKD) who experienced repeated arteriovenous fistula (AVF) thromboses and multiple catheter-related bloodstream infections, ultimately exhausting all viable vascular access sites for hemodialysis (HD). Despite prior abdominal surgeries, the patient underwent successful PD catheter insertion following femoral catheter-related sepsis and fungemia. Although initial PD catheter malposition was observed, it was corrected surgically, and PD was initiated, leading to full clinical recovery. This case highlights the potential role of PD as a rescue therapy, even in patients with relative contraindications and no remaining HD access options. It underscores the importance of reconsidering the feasibility of PD in high-risk patients when vascular access is no longer available.

## Introduction

End-stage kidney disease (ESKD) often requires long-term renal replacement therapy. Globally, hemodialysis (HD) is the predominant modality, with over 80% of chronic dialysis patients receiving in-center HD in most countries [1]. However, successful HD depends on the availability of functional vascular access, such as an arteriovenous fistula (AVF), a graft, or a tunneled central venous catheter (CVC). In some patients, repeated vascular access failure and catheter-related bloodstream infections may lead to the complete exhaustion of viable vascular access, posing a critical therapeutic dilemma for clinicians [2,3].

Peritoneal dialysis (PD) is a valuable alternative when HD access options are exhausted, but its use may be limited in patients with prior abdominal surgeries, intra-abdominal adhesions, or previous kidney transplantation, anatomical factors known to increase the risk of catheter malfunction or PD failure [4]. Although such histories have traditionally been viewed as relative contraindications, current guidelines support careful evaluation and individualized decision-making rather than outright exclusion, especially when no other modalities are viable [5].

This case report presents a patient with exhausted HD access and multiple prior abdominal interventions, including nephrectomy, kidney transplantation, and hernia repair, who underwent a successful transition to PD despite not being considered an ideal candidate. The case illustrates how PD can serve as a rescue modality even in challenging clinical scenarios.

## Case presentation

A 64-year-old male with ESKD secondary to hypertensive nephrosclerosis was admitted due to infection of a tunneled Hickman CVC. On admission, he was febrile (38.5°C), with elevated inflammatory markers (CRP 66 mg/L), and blood cultures grew coagulase-negative *staphylococci*. His past medical history included hypertension, emphysema, ischemic cardiomyopathy with a left ventricular ejection fraction of 35%, bilateral inguinal hernia repair, and right nephrectomy for renal cell carcinoma. He had previously started HD 19 years ago using a left forearm AVF and received a cadaveric kidney transplant 16 years ago. Due to graft failure, HD was resumed six years later.

Over the years, multiple AVF creation attempts were unsuccessful due to thrombosis despite anticoagulant therapy. The patient subsequently became dependent on tunneled CVCs via various access sites, including both internal jugular veins and femoral veins. Repeated catheter-related bloodstream infections, including previous episodes of Acinetobacter spp. and coagulase-negative *staphylococcal* sepsis, complicated his course. One CVC was inadvertently placed into the transplanted kidney vein and required removal. Another attempt resulted in vascular injury and hematoma in the femoral region, necessitating surgical repair of the femoral artery and vein.

**Figure 1:**
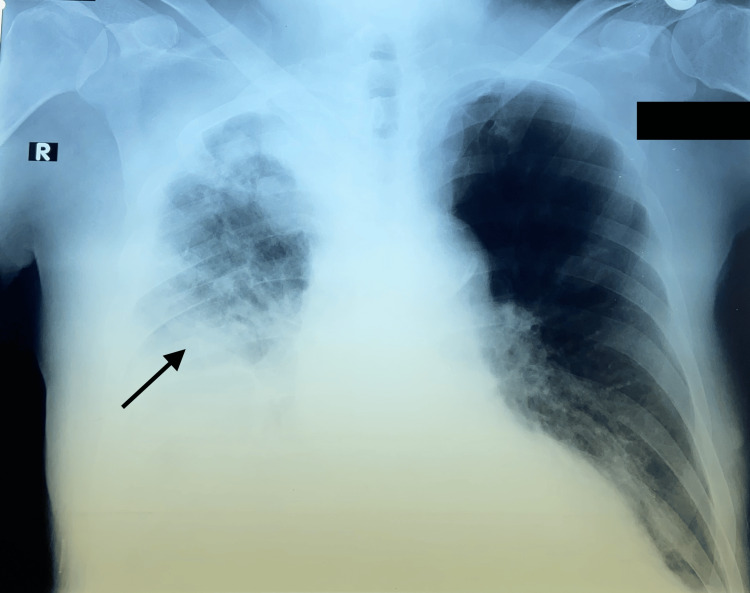
Anterior-posterior view chest X-ray showing right-sided pneumonia with pleural effusion (black arrow).

After the most recent CVC placement in the left femoral vein, the patient developed recurrent infection with persistent fever, pneumonia, and fluid overload (Figure 1).

Despite targeted antimicrobial therapy, his condition worsened. Blood cultures later revealed *Candida spp.*, and he required oxygen therapy and inotropic support. Given the lack of viable vascular access and poor clinical response, the nephrology and vascular surgery teams opted to initiate PD, despite the patient's history of prior abdominal surgeries, which rendered him a high-risk candidate.

**Figure 2:**
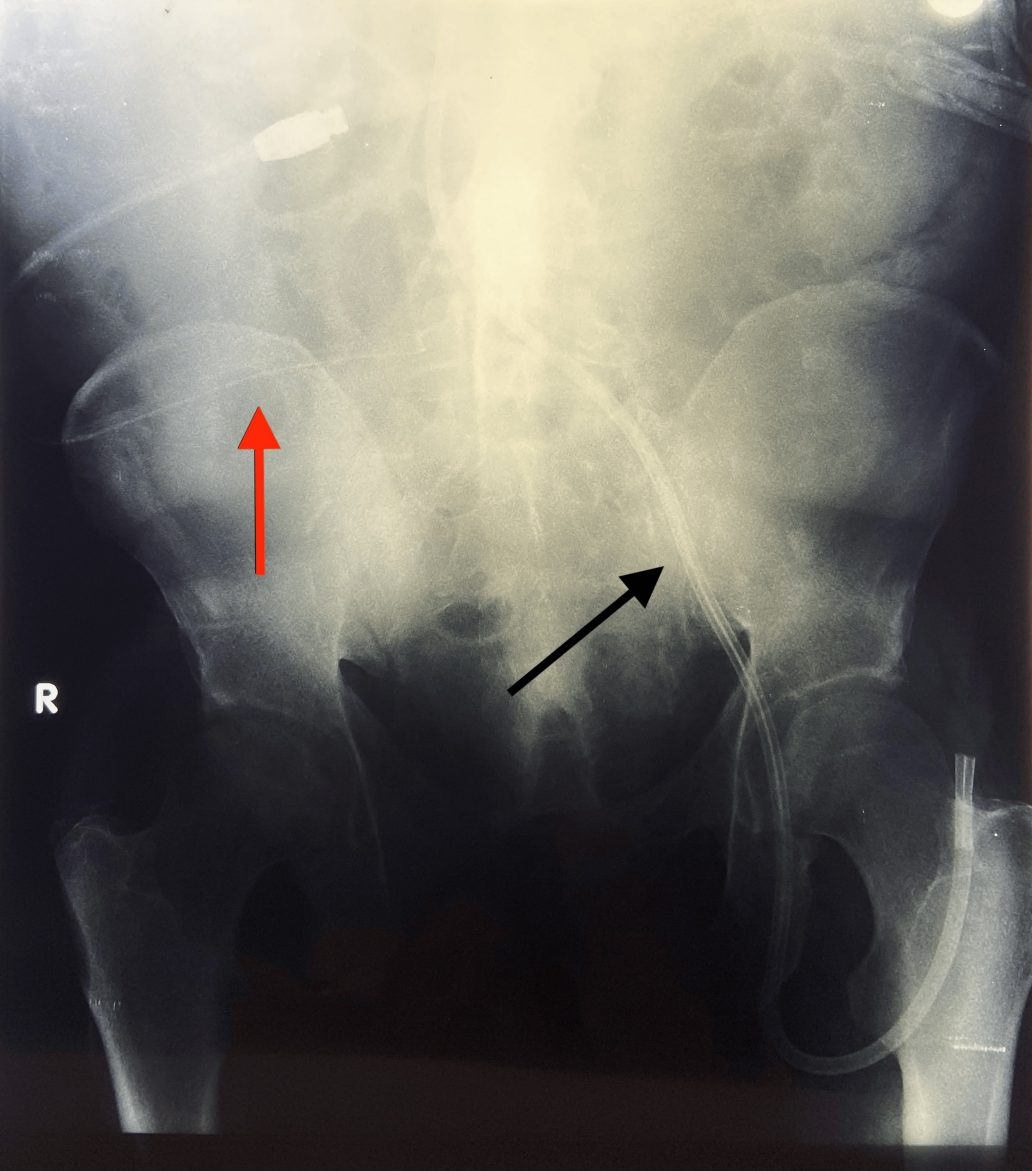
Native X-ray of the abdomen showing the PC (red arrow) and infected Hickman CVC (black arrow). PC: Peritoneal catheter; CVC: Central venous catheter.

The initial PD catheter was malpositioned and required replacement (Figure 2).

**Figure 3:**
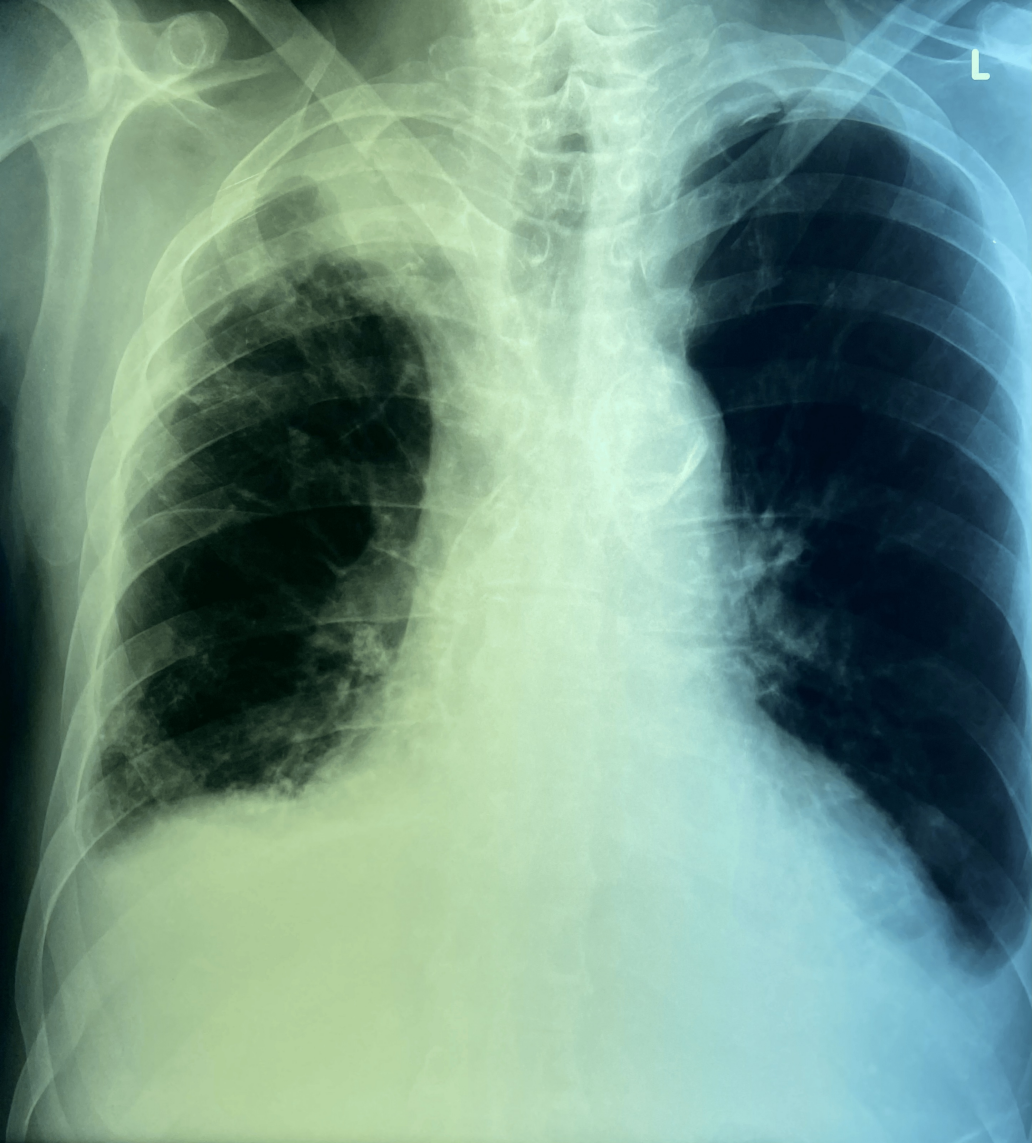
Anterior-posterior view chest X-ray showing regression of pneumonia.

Ten days after successful placement of the second PD catheter, PD was initiated, and the infected Hickman CVC was removed. The patient’s clinical status improved significantly, with defervescence, radiographic resolution of pulmonary infiltrates (Figure 3), and normalization of inflammatory markers (Table 1).

**Table 1:** Laboratory values during hospitalization. A hyphen (-) indicates that the laboratory value was not measured at the corresponding time point.

Laboratory Values	Hospital Admission	Day Before Hickman CVC Removal	Day Before Discharge	Normal Range
Leukocytes (×10⁹/L)	6.4	9.4	3.4	3.4-9.7
Erythrocytes (×10¹²/L)	3.46	2.6	3	4.34-5.72
Hematocrit (L/L)	0.31	0.23	0.29	0.41-0.53
Hemoglobin (g/L)	106	78	90	122-157
Platelets (×10⁹/L)	180	118	223	150-450
Glucose (mmol/L)	3.9	3.1	4.3	3.9-6.1
Serum Urea (mmol/L)	16.4	32.2	14.6	2.5-7.5
Serum Creatinine (μmol/L)	739	1223	942	45-84
C-reactive Protein (mg/L)	66.1	379	26.7	< 5.0
Procalcitonin (ng/mL)	32.1	330	0.34	< 0.5
Fibrinogen (g/L)	7.4	-	3.3	2.0-4.0
D-dimer (mg/L)	1.95	3.81	1.62	< 0.5
Activated Partial Thromboplastin Time (s)	33.6	41.3	-	22.0-32.0
International Normalized Ratio (INR)	1.09	1.28	-	0.8-1.2
Total Protein (g/L)	57	51	46	62-81
Albumin (g/L)	37	26	24	34-55
Calcium (mmol/L)	2.25	2.1	2.07	2.15-2.65
Phosphorus (mmol/L)	1.71	3.33	1.7	0.80-1.55
Sodium (mmol/L)	141	133	142	135-148
Potassium (mmol/L)	5.3	6.3	3.5	3.5-5.1
Aspartate Transaminase (U/L)	24	111	18	0-37
Alanine Transaminase (U/L)	14	44	15	0-41
Alkaline Phosphatase (U/L)	59	198	114	40-120
Gamma-Glutamyl Transferase (GGT) (U/L)	14	96	40	0-55
Lactate Dehydrogenase (LDH) (U/L)	630	1107	451	220-460

He was discharged in stable condition, with no further infectious complications.

## Discussion

Central vein stenosis, which is common after multiple CVCs, can impair AVF function by limiting outflow, increasing recirculation, and prolonging bleeding [6]. Additionally, hypercoagulable states should be suspected in patients with repeated access failures without clear anatomical causes [7]. Studies have identified thrombophilic abnormalities, such as Factor V Leiden or antiphospholipid syndrome, in such cases [8].

When no vascular access remains for HD, several last-resort strategies may be considered. The HeRO graft, a hybrid graft-catheter device, can bypass central venous occlusions but requires partial central patency to be effective. More extreme options include trans-lumbar or trans-hepatic catheters, which access the inferior vena cava via the abdomen or liver. Though technically feasible, these approaches carry significant risks, including hemorrhage, organ injury, and infection, and demand specialized expertise [6,9].

This case challenges the traditional reluctance to use PD in patients with prior abdominal surgeries. Although adhesions and hernia repairs have been considered relative or even absolute contraindications, growing evidence suggests these concerns may be overstated. A large North American registry analysis (2011-2020) found no significant increase in PD catheter failure or complications in patients with a history of prior abdominal surgery [10]. Even those with multiple procedures did not show cumulative risk. This perspective aligns with other experiences where PD was successfully performed in patients with conditions long considered incompatible with PD, including prior hemicolectomy, colostomy, extensive adhesions, and even congenital anomalies such as spina bifida [11]. These findings reinforce that many traditional contraindications are not absolute and that interdisciplinary collaboration, advanced catheter techniques, and individualized planning can enable PD in highly complex scenarios [11].

Furthermore, the literature suggests that PD should be reconsidered in “last-resort” scenarios. For patients with no vascular access, PD can avoid long-term catheter-related complications and serve as a bridge to future treatments, such as kidney re-transplantation, while maintaining dialysis adequacy and quality of life [12].

## Conclusions

This case demonstrates that PD remains a viable and potentially lifesaving option, even in patients traditionally considered poor candidates due to prior abdominal surgeries and exhausted vascular access. With careful patient selection, interdisciplinary planning, and modern interventional techniques, PD can be successfully implemented in high-risk scenarios, challenging outdated contraindications and expanding treatment options in complex ESKD cases. Given the growing body of evidence and our clinical experience, PD should be considered not only as a salvage option but also as a proactive strategy in patients at high risk for vascular access exhaustion.
